# Placenta Previa Percreta: A Case Report of Successful Management via Conservative Surgery

**DOI:** 10.1155/2013/702067

**Published:** 2013-01-17

**Authors:** Silvia Canonico, Maurizio Arduini, Giorgio Epicoco, Giuseppe Luzi, Saverio Arena, Graziano Clerici, Giuseppe Affronti

**Affiliations:** S.C. Ostetricia e Ginecologia, Azienda Ospedaliera S. Maria della Misericordia, 06132 Perugia, Italy

## Abstract

Placenta percreta is one of the most serious complications of placenta previa and is frequently associated with severe obstetric hemorrhage usually necessitating hysterectomy. We present a case of placenta previa percreta diagnosed by ultrasound and magnetic resonance imaging techniques, in which we accomplished conservative management of postpartum hemorrhage. The management we propose includes the following steps: preventive catheterization of the descending aorta via transhumeral access; Stark cesarean delivery; uterotonics drugs; Affronti endouterine square hemostatic sutures; intrauterine application of Bakri balloon and partial filling with 100 mL of normal saline; B Lynch suture, hysterorrhaphy, and filling a Bakri balloon with up to 500 mL of normal saline; reversible radiological embolization; and/or surgical ligation of the uterine arteries. The bleeding stopped following placement of Affronti sutures combined with external (B-Lynch suture) and internal (Bakri balloon) uterine compression. Our experience indicates that this conservative method can be considered an option in the management of selected cases of pregnancy at high risk for intrapartum hemorrhage.

## 1. Introduction

Placenta accreta refers to a placenta that is abnormally adherent to the uterus [1]. There are three main entities (accreta, increta, and percreta), which are defined by histological degree of placental invasion into the myometrium [1]. Placenta accreta is one of the most serious complications of placenta previa and is frequently associated with severe obstetric hemorrhage, usually necessitating hysterectomy if medical therapies have failed.

We present a case of placenta previa percreta diagnosed by ultrasound and magnetic resonance images, in which we accomplished conservative management of *postpartum* hemorrhage. 

## 2. Case Report

**Figure 1:**
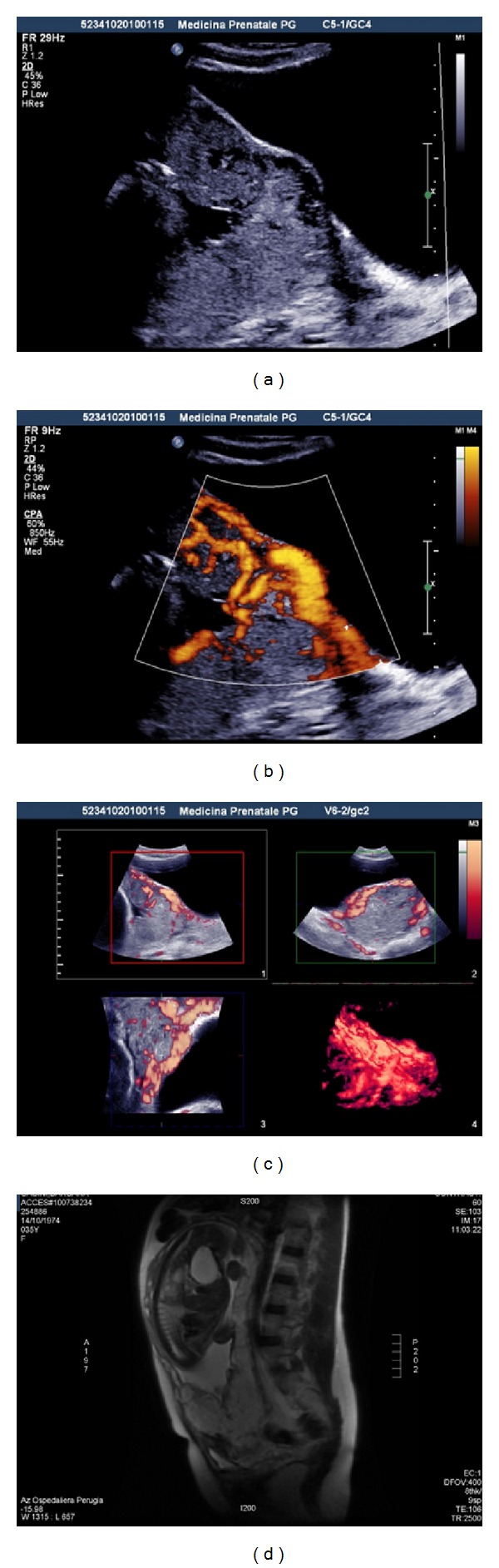
Ultrasonographic-RMN diagnosis of placenta previa accrete. (a) 2D transabdominal ultrasound showed the absence of placental- miometral interface, the uterine wall being undistinguishable from the placenta, and the presence of multiple vascular intraplacental lacunae “Swiss cheese placental appearance,” (b) 2D color Doppler ultrasound revealed an extensive vascularity along the anterior portion of the lower uterine segment and appears to extend up to and around the bladder, (c) 3-dimensional power Doppler showed the presence of numerous retroplacental vessel, and (d) pelvic magnetic resonance (MR) confirmed the ultrasound diagnosis of placenta previa accreta with partial wall bladder invasion.

A 33-year-old woman (gravida 3, para 1) was referred to our hospital due to abnormal placentation diagnosed during a routine sonographic examination at 27 weeks of gestation. She had a history of prior cesarean section due to placenta previa, five years earlier. On admission, the patient presented spotting without abdominal pain. An urgent abdominal ultrasound examination showed a viable fetus with appropriate biometrical parameters and normal amniotic fluid, while transvaginal Eco-Doppler images suggested the diagnosis of placenta previa accreta (Figure 1).

Careful evaluation of the placenta with pelvic noncontrast magnetic resonanceimaging (MRI) confirmed the ultrasound diagnosis (Figure 1). The patient's urinalysis was negative for blood, and cystoscopy did not reveal bladder invasion. The patient was made aware of the potential obstetric complications. An elective cesarean delivery was planned at 35 weeks of gestation.

On the scheduled day of delivery, a preliminary prophylactic catheterization of the descending aorta by transhumeral access was performed. After opening the abdominal wall, intra-abdominal inspection showed the presence of multiple large vessels at level of the lower uterine segment, under the peritoneum and in the area of the bladder. An initial displacement of the bladder was performed and subsequently a transverse uterine incision was made above the lower uterine segment in order to avoid placental bed. A healthy neonate weighing 2,380 g was delivered. In order to minimize uterine bleeding, a Satinsky atraumatic clamps were initially positioned around the uterine arteries. Spontaneous placental extraction failed, the placental tissue was manually removed, and the placental site was inspected by instrumental review. Five Affronti hemostatic sutures were applied in the area of bleeding at the site of the placental bed using 1.0 vicryl [2]. Later a B-Lynch compression suture was prepared (2.0 coated vicryl) and a Bakri balloon (Cook Medical, Bloomington, IN, USA) was inserted into the uterus through the hysterotomy site and filled with 100 mL of normal saline [3]. Finally, the abdomen was closed using a regular technique. The total blood loss was 1.500 mL. Intraoperative evaluation of CBC revealed a hematocrit 25.7% and hemoglobin 5,9 g/L. Intraoperative allogeneic red blood cells (1050 mL) and free-frozen plasma (1,400 mL) were transfused. The patient was cared for in the intensive care for one day. The postoperative course was uneventful, and the patient was discharged on day 7 in good conditions. 

## 3. Discussion

The incidence of placenta accrete has increased from approximately 0.8/1000 deliveries in the 1980s to 3/1000 deliveries in the past decade [4, 5]. The early diagnosis of placenta previa is usually made by ultrasound [6], although in recent years there has been an interest in the use of MR imaging [6, 7]. Despite the early and accurate prenatal diagnosis, hysterectomy remains the most common surgical procedure in cases of PPH for placenta previa accreta [8]. Nowadays, conservative interventions are recommended before radical procedure in order to minimize surgical complications and preserve fertility. The conservative options for PPH included uterotonics drugs, external compression with uterine sutures (B-Lynch, Hayman, Cho), intrauterine packing (Bakri balloon), and selective devascularization by ligation or embolization of the uterine artery [4, 9–11].

Nelson and O'Brien reported that placing an intrauterine Bakri balloon in conjunction with the B-Lynch uterine compression suture defined as “uterine sandwich” was successful in treating uterine atony [12].

We report on a successful outcome of our conservative surgical protocol in a case at high risk of PPH for placenta previa accreta percreta.

The first step of our conservative protocol is the preliminary prophylactic catheterization of the descending aorta by transhumeral or transfemoral access. Subsequently, several surgical treatments were combined. Initially endouterine square hemostatic Affronti's suture was applied to the area of bleeding at the site of the placental bed. The sutures are square in shape measuring approximately 2 cm and penetrate from the endometrium to the myometrium without extending beyond the uterine serosa. Tying the two ends of the Affronti suture retracts the myometrial fibers and causes a reduction in bleeding [2]. 

Subsequently the “sandwich technique,” combined with endouterine Affronti's sutures, induced a rapid hemostasis at the site of placental insertion.

In conclusion, our experience indicates that conservative methods can be considered as an option in the management of selected cases of pregnancy at high risk for intrapartum hemorrhage.
